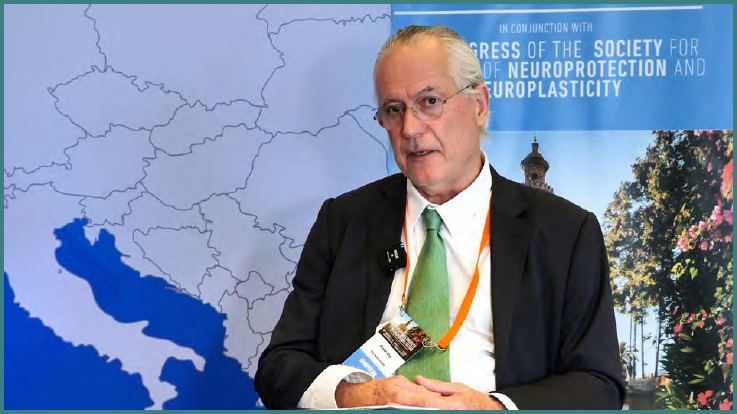# Interview with Dr. Pieter Vos - 8^th^ European Congress on Neurorehabilitation in conjunction with the 20^th^ Congress of the Society for the Study of Neuroprotection and Neuroplasticity

**DOI:** 10.25122/jml-2026-1006

**Published:** 2026-04

**Authors:** Stefana-Andrada Dobran, Alexandra Gherman

**Affiliations:** 1 RoNeuro Institute for Neurological Research and Diagnostic, Cluj-Napoca, Romania; 2 Sociology Department, Babes-Bolyai University, Cluj-Napoca, Romania

Interviewee: Dr. Pieter Vos

Interviewer: Ms. Stefana-Andrada Dobran

Dr. Pieter Vos is a neurologist at Slingeland Hospital in the Netherlands, specializing in traumatic brain injury (TBI), and has previously worked at the Department of Neurology at Radbound University Medical Center. For decades, he has focused on the clinical, biochemical, and genetic factors that influence neuroplasticity and recovery after TBI. A leading figure in European neurotrauma, he currently co-chairs the European Academy of Neurology's Task Force on Mild TBI (until 2026) and serves on its Neurotraumatology panel. He has also led major clinical studies and contributed to international treatment guidelines.

**S.D.: Hello, dear Dr. Pieter Vos. Welcome to the 8^th^ European Congress on Neurorehabilitation (ECNR) in conjunction with the 20^th^ Congress of the Society for the Study of Neuroprotection and Neuroplasticity. The ECNR brings together the scientific and clinical communities. What do you believe is the unique role that it plays in bridging the gap between research and daily patient care in neurorehabilitation?**

P.V.: I believe that the unique role of the EFNR is that it brings together a multidisciplinary group of persons. As you might know, many diseases that require neurorehabilitation require many specialists. And I think it's very good and interesting to have these meetings with so many people from different disciplines: neurologists, rehabilitation specialists, physiotherapists, psychologists, neuropsychologists, etc.

**S.D.: Considering your medical specialty, what future developments do you envision for the complex multidisciplinary field of neurorehabilitation?**

P.V.: My medical specialty is neurology. I'm a neurologist. In the older days, I did a lot of research, but in the last decade, my work has been dedicated to clinical neurology. From the clinical perspective—where you see a lot of acutely diseased patients, like those with stroke or traumatic brain injury—I think that for the future, we need to continue the multidisciplinary approach and provide good education, perhaps with better guidelines. We need guidelines that people can engage with so that they will adhere to them. And I think that the European Congress or the European Federation can play an important role in that field.


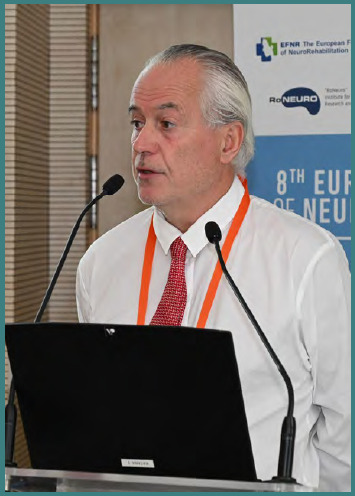


**S.D.: What emerging trend or technology are you most excited about in the field?**

P.V.: The obvious answer will be AI, artificial intelligence. I really think that it's going to play an important role. However, on the other hand, I think it will be important for medical specialists—whether doctors, physiotherapists, or physician assistants—to keep playing their role, although their specific roles may evolve in this respect. I spoke to a colleague of mine who is convinced that in the next few years, because of the enormous amount of data that is available, the role of physicians, neurologists, or other specialists will become smaller. I am, of course, excited about these technological developments, but we have to see how that works out.

**S.D.: What is, in your perspective, the most challenging future development in neurorehabilitation and how can the EFNR come closer to that endeavor?**

P.V.: Of course, you can again mention the role of AI or robotics, or the techniques that are developed. For instance, amyotrophic lateral sclerosis (ALS) is not just a research topic. As a neurologist, I have seen many patients with ALS, and there, for instance, is a development where you can place devices on the brain, enabling severely diseased patients to still communicate with others. So these are, of course, developments that are really changing the field, although it's still in its beginning phase.

The same is true for spinal cord injury, where there have been publications on devices and interfaces that aim to enable spinal cord-injured patients, for instance, to walk again or to use an arm or a hand.

**S.D.: And regarding the role of the EFNR?**

P.V.: Yes, that's of course an important issue. The EFNR has not existed for many years, so it's an emerging organization. I'm very enthusiastic about it. I'm from the Netherlands. In the Netherlands, we have diverse specialties, so rehabilitation is a different specialty than neurology. We're talking here about neurorehabilitation, so I'm not aware how it is in all countries, but I think that the EFNR, as an organization specialized in neurorehabilitation, can play an important role. For instance, it can serve rehabilitation physicians who are more focused on general rehabilitation and want to learn about or see the research developments in neurorehabilitation. And also for neurologists, or neurologists specialized in rehabilitation, I think the EFNR can be an important platform—either via online education, education at congresses, or by stimulating research and collaboration among the different specialties.

**S.D.: As co-chair of the European Academy of Neurology (EAN) Task Force on mild TBI, what is the most important change that you are advocating for?**

P.V.: It's true, I'm co-chair of the task force that will try to construct a guideline under the umbrella of the European Academy of Neurology on mild traumatic brain injury or concussion, as it is also called. I will be chairing together with my colleague Luca Sebastianelli from Italy. It's a very interesting task, and we are, in this respect, highly excited that we are able to put a multidisciplinary group together consisting of neurologists, neurosurgeons, neuropsychologists, rehabilitation specialists, psychiatrists, and, of course, members from the patient community.


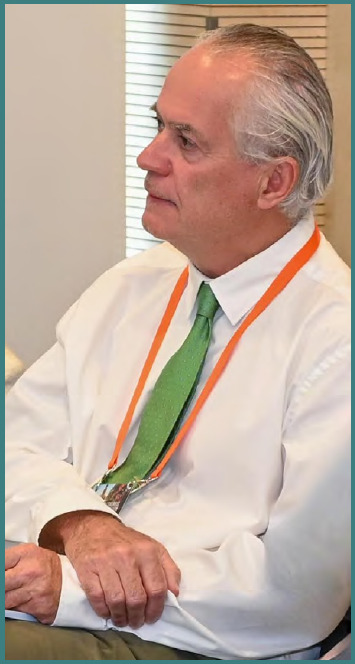


We are about to start, so I cannot say whether we will go this or that way. The main point I'm advocating for is that we ask the right questions. With those right questions, which we can then, of course, develop in a scientific way, the guideline will ultimately help the patients, but also doctors and other people who are involved in caring for patients in the acute phase at the hospital—when patients are admitted or brought to the emergency department—as well as in the chronic phase, which is a topic in itself.

What happens to those patients with mild traumatic brain injury who, in general, almost all survive and have a small chance of serious, life-threatening complications—for instance, an epidural or subdural hematoma? This is only a small minority, but mild traumatic brain injury accounts for enormous numbers. Many people every year sustain a mild traumatic brain injury and of those a small but significant portion develops chronic complaints. I hope that we really can ask the right questions to come up with a practical guideline that will help everybody.